# Loss of Progranulin Expression Decreases NLRP3 Inflammasome-Mediated Inflammation and Enhances Bone Anabolism

**DOI:** 10.21203/rs.3.rs-9441627/v1

**Published:** 2026-05-06

**Authors:** Vikrant Piprode, Liping Wang, Gursimar Kohli, Petronela Nicolae, Yue Wang, Chun Wang, Gabriel Mbalaviele, Mary Nakamura, Robert Nissenson

**Affiliations:** San Francisco VA Medical Center/University of California San Francisco; San Francisco VA Medical Center/University of California San Francisco; San Francisco VA Medical Center; San Francisco VA Medical Center/University of California San Francisco; Skaggs School of Pharmacy, University of Colorado; Washington University School of Medicine; Washington University School of Medicine; UCSF; San Francisco VA Medical Center/University of California San Francisco

**Keywords:** Progranulin, macrophages, inflammation, NLRP3, inflammasomes, IL-1β, arthritis, iPTH

## Abstract

Progranulin (PGRN), a glycosylated protein, is expressed in most tissues, including bones, and its level is elevated in the serum and joints of individuals with inflammatory bone loss disorders such as rheumatoid arthritis (RA). Previously, using global and macrophage-specific *Grn* deletion mice, we demonstrated that loss of PGRN protects against age-related bone loss selectively in females, suggesting a sex-dependent role for macrophage-derived PGRN in skeletal homeostasis. Here, we investigated the role of PGRN in the regulation of macrophage-mediated inflammation and bone formation. Immune-phenotyping revealed that *Grn*^−/−^ vs. WT mice exhibited higher percentages of Mac2^Hi^ subsets in both sexes. Transcriptomic analysis of Mac2^Hi^ vs. Mac2^Lo^ from WT mice showed reduced expressed expression of *Nlrp3* and *Il1β* at baseline in both sexes. Further, *Grn*^−/−^ vs. WT M-CSF-dependent macrophages (BMMs) revealed decreased expression of *Nlrp3* and *Il1β* following LPS challenge in vivo in both sexes. Consistent with this, *Grn*^−/−^ mice displayed markedly reduced IL-1β production in serum and paw joints, and attenuated bone erosion in the STA-induced RA model, indicating altered NLRP3 inflammasome signaling in *Grn*^−/−^ mice. Notably, PGRN deficiency enhances the osteoanabolic capacity of macrophages: female *Grn*^−/−^ BMMs potentiated osteogenic differentiation of mesenchymal stem cells, and *in vivo*, *Grn*^−/−^ females exhibited higher trabecular bone formation in response to intermittent PTH. Collectively, PGRN deficiency in BMMs is negatively associated with *Nlrp3* expression and IL-1β production and causes reduced inflammation and bone erosion in mice subjected to STA-induced RA. Furthermore, PGRN limits the bone-anabolic action of PTH in a female sex-specific manner.

## INTRODUCTION

Progranulin (PGRN), a glycoprotein encoded by the *Grn* gene, is constitutively expressed in different tissues, including epithelia, particularly in skin, gastrointestinal tract, reproductive system, immune cells, specific neurons, microglia, and adipose tissue ^1,2^. Intracellularly, PGRN is in lysosomes, where it regulates protein trafficking, functions as a molecular co-chaperone and helps acidification of lysosomes ^3^, and as secretory protein, PGRN acts as a growth factor ^4–7^.It binds to several cell surface receptors such as tumor necrosis factor-α receptor 1 and 2 ^8^, Sortilin Related VPS10 Domain Containing Receptor 2 ^9^, EGFR ^10^, and Ephrin A2 ^11^, to mediate differential cellular functions. Consequently, PGRN plays a pivotal role in embryogenesis, inflammation, wound healing, and tumorigenesis ^1^. In humans, a heterozygous loss-of-function (LOF) mutation in *Grn* causes Frontotemporal lobar dementia (FTLD/FTD), while homozygous LOF mutation leads to neural ceroid lipofuscinosis type 11 (NCL11), characterized by vision loss, dementia and epilepsy ^12^.

PGRN has been widely implicated in immune regulation, particularly in macrophage biology. It inhibits LPS-induced M1 macrophage polarization of RAW264.7 cells, bone marrow-derived macrophages (BMMs), and THP-1 cells ^13^. Consistent with its anti-inflammatory effects, *Grn*^−/−^ mice undergo spontaneous osteoarthritis and exhibit exacerbated inflamed joints in collagen-induced arthritis (CIA) model ^14,15^. Moreover, PGRN enhances M2 macrophage polarization of BMMs and RAW264.7 as shown by a decrease in CD86 expression (M1 marker) and increase in CD206 expression (M2 marker) ^16^. *In vivo*, recombinant PGRN increased CD206 + macrophages in unilateral ureteral obstruction (UUO) model ^17^. Furthermore, *Grn*^−/−^ mice were protected mice from endotoxic shock following LPS injection and showed reduced lung injury ^18^. All these studies indicated an anti-inflammatory role of PGRN. Conversely, higher serum PGRN levels are reported in RA, OA and Type 2 Diabetes (T2D) individuals. In mice, PGRN mediates high fat diet-induced insulin resistance via IL-6 production in adipose tissue ^19^. Similarly, in severe SLE individuals, higher serum PGRN levels positively correlate with higher serum IL-6 and TNFα ^19^. Further, PGRN stimulates secretion of IL-8 by epithelium cells in Multiple Sclerosis (MS), which acts as chemotactic factor for neutrophils and monocytes ^19^. Thus, PGRN is one of those molecules whose imbalance in levels brings out different outcomes depending on the cell type and the diseases being examined, also known as Progranulinopathies, which include autoimmune diseases, metabolic, musculoskeletal and cardiovascular diseases. The dual function of progranulin: as a pro- and an anti-inflammatory molecule, depending on the cellular context is elaborated in the review by Huang et al ^19^.

Previously, we demonstrated that global deletion of *Grn* protects female but not male mice from aging-induced bone loss. A similar phenotype was observed in *Cx3Cr1*^*Cre*^; *Grn*^*f/f*^ female mice, suggesting a critical role of macrophage-derived PGRN in regulating bone homeostasis in female mice ^20^. Macrophages impact bone health, indirectly via inflammation, and directly act as osteoclast precursors, and as osteomacs to enhance or inhibit bone formation functions of osteoblasts depending on the macrophage phenotype ^21^. Additionally, PGRN protects from TNF-ɑ-induced inhibition of osteoblast differentiation and mineralization ^22^, underscoring its dual role in bone remodeling.

In FTD individuals with GRN mutations, neuroinflammation due to microglia activation produces inflammatory cytokines including IL-1β and IL-18. These two cytokines are products of NLRP3 inflammasome cascade ^23^. NLRP3, the NOD-, LRR-, and pyrin domain-containing 3 inflammasome, is a well-known innate immune pathway and is activated by DAMPs and PAMPs. Consequently, caspase-1 is activated which produces bioactive IL-1β and IL-18 to induce inflammatory cell death called pyroptosis ^24^. These observations suggest an association of PGRN with NLRP3 inflammasome cascade.

In the present study we investigated the role of PGRN in influencing macrophage functions which ultimately impact bone health. We find that *Grn*^−/−^ BMMs are less inflammatory than the PGRN-replete BMMs. Mechanistically, *Grn*^−/−^ BMMs exhibit down-regulation of *Nlrp3* and *Il1β* expression and show poor response to LPS-induced inflammation and M1 polarization. As a result, *Grn*^−/−^ mice show significant alleviation of inflammation in response to serum transfer-induced arthritis. Moreover, *Grn*^−/−^ female BMMs mice show higher bone anabolic activity in vitro and in response to PTH administration, indicating that PGRN limits the anabolic action of PTH therapy. Mechanistically, our bulk RNA-seq data shows downregulation of G_i_ signaling in the *Grn*^−/−^ osteoblasts, which in part, can contribute to enhanced bone formation via activation of Gs signaling in response to PTH. In summary, these results suggest that PGRN expression is associated with NLRP3-inflammasome-mediated inflammation and suppression of bone anabolic functions of macrophages.

## RESULTS

### Freshly isolated macrophages from Grn ^−/−^ are Mac2^hi^ subset with decreased inflammatory phenotype

**Figure 1:**
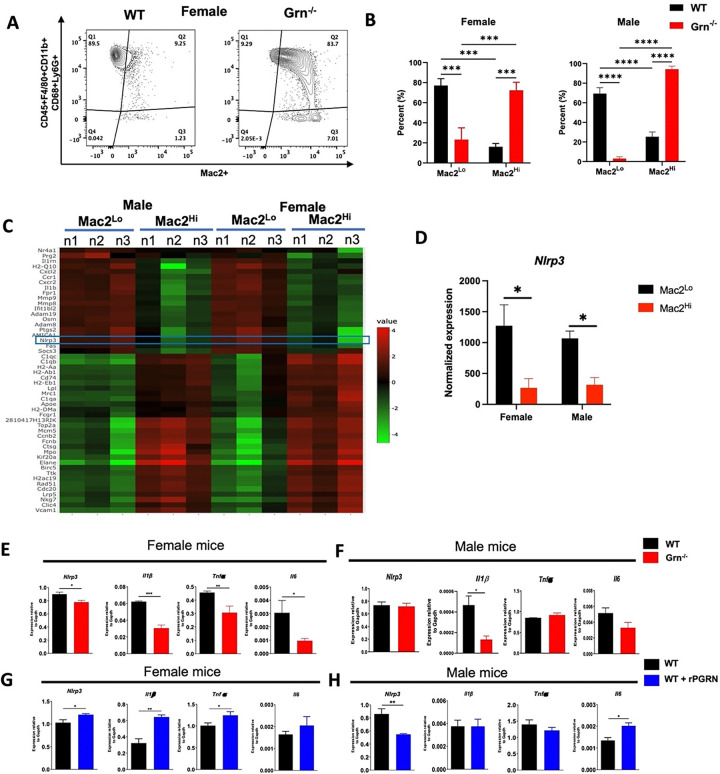
Freshly isolated macrophages from *Grn*^−/−^ mice are Mac2^Hi^ subset with decreased inflammatory phenotype **A,** Representative data from the flow cytometric analysis of the live bone marrow macrophages (CD45^+^F4/80^+^CD11b^+^Ly6G^+^Ly6C^+^cells) from 7-months-old female *Grn*^−/−^ and littermate WT mice. The macrophages consisted of Mac2^Lo^ and Mac2^Hi^ subsets. The gates were determined through fluorescence minus one (FMO) controls. **B,** Distribution of Mac2^Lo^ and Mac2^Hi^ macrophage subsets (% of total analyzed cells) in *Grn*^−/−^ and WT bone marrow from both the sexes. These subsets were sorted from WT mice and subjected to NanoString-based transcriptomics. **C**, Heatmap showing differential expression of genes from n=3 mice/ group (n1, n2, n3). **D**, normalized expression of *Nlrp3* in both females and males BMMs. The intrinsic expression of inflammatory genes including *Nlrp3*, *Il1β*, *Tnfα*, and *Il6* was assessed by qPCR in WT and *Grn*^−/−^. M-CSF dependent bone marrow-derived macrophages (BMMs) in **E**, female and **F**, male cohorts. WT BMMs from both sexes were treated with rPGRN at 500 ng/mL for 24 hrs and expression of these inflammation-related genes was assessed by qPCR. Data in **B** are presented as mean ± SEM (n=4 for each genotype in female cohort; and n=6 WT and n=4 *Grn*^−/−^ in male cohort), and was analyzed using Two-way ANOVA using uncorrected fisher LSD correction. Data in **D-H**, are presented as mean ± SEM of n=3 for each genotype in both the sexes and were analyzed using non-parametric t-test.

As reported, female mice with PGRN deficiency in macrophages (*Cx3Cr1*^*Cre*^; *Grn*^*f/f*^ mice) are protected from aging-induced bone loss ^20^. Thus, we began characterizing the freshly isolated bone marrow macrophages from WT and *Grn*^−/−^ mice of both sexes ^25^. We found similar percentages of total macrophages in *Grn*^−/−^ and WT mice in both sexes expressing CD11b^+^/CD45^+^/F4/80^+^/Ly6G^+^/Ly6C^+^. Interestingly, in female *Grn*^−/−^ mice, ~ 72.35% of these macrophages were Mac2 high (Mac2^Hi^, positive for Mac2) and only ~ 23.39% were Mac2 low (Mac2^Lo^, negative for Mac2). Conversely, in female WT mice, ~ 16.18% were Mac2^Hi^ and ~ 77.2% were Mac2^Lo^ (Fig. 1A and 1B). Male *Grn*^−/−^ mice exhibited ~ 94.27% Mac2^Hi^ and ~ 3.09% Mac2^Lo^ macrophages, and male WT showed ~ 25.39% of Mac2^Hi^ and ~ 69.4% Mac2^Lo^ (Fig. 1B). Furthermore, NanoString-based transcriptomics on FACS-sorted Mac2^Hi^ subsets displayed enrichment of cell cycle genes likely indicating a higher proliferation rate. Contrarily, Mac2^Lo^ macrophages showed higher expression of inflammation-related genes, notably, *Nlrp3* and *Casp1* (Fig. 1C, 1D). As activation of the NLRP3 inflammasome pathway is sufficient to polarize macrophage towards inflammatory M1 type, we focused on understanding NLRP3 inflammasome signaling in the macrophages. As *Grn*^−/−^ have higher % of Mac2^Hi^ subset, we assessed NLRP3 inflammasome pathway in M-CSF dependent bone marrow-derived macrophages (BMMs) from WT and *Grn*^−/−^ mice. We found that female *Grn*^−/−^ vs. WT BMMs displayed reduced expression of *Nlrp3*, *Il1ß*, *Tnfa* and *Il6* (Fig. 1E). However, male BMMs showed decreased *Il1ß* with other genes remained unchanged (Fig. 1F). Additionally, female WT BMMs treated with rm PGRN for 24 hrs greatly enhanced the expression of *Nlrp3* and *Il1ß* genes but not in males (Fig. 1G and 1H). These findings suggest an upstream regulatory role of PGRN in the expression of *Nlrp3* pathway genes in female mice.

### Grn ^−/−^ mice exhibit a suppressed inflammatory response to LPS administration

**Figure 2:**
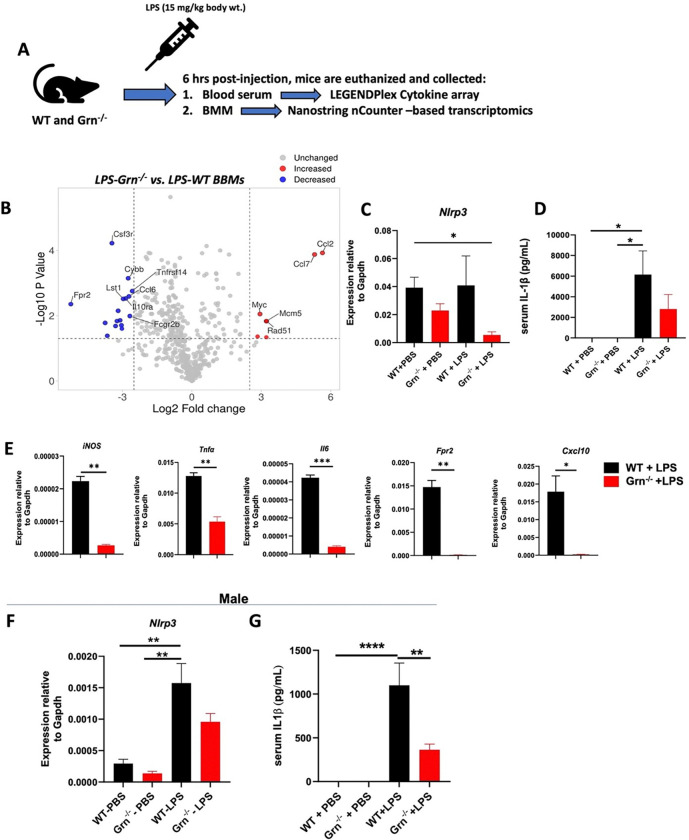
*Grn*^−/−^ mice display attenuated LPS-induced NLRP3-inflammasome associated inflammatory responses **A**, Female and male WT and *Grn*^−/−^ mice of 6–8 -months-old were i.p. administered with LPS (15 mg/kg body wt). After 6 hrs, M-CSF-denependent macrophages were preparaed and total RNA was isolated and subjected to Nanostring-based transcriptomics which was analyzed and a **B**, volcano plot showing the expression profiles of myeloid innate immune genes in red (up-regulated) and blue dots (down-regulated), assessed by Nanostring in the *Grn*^−/−^-LPS vs. WT-LPS female mice. Bar graphs showing **C**, *Nlrp3* expression as assessed by qPCR, and **D**, serum IL-1β was evaluated using LEGENDplex^™^ array. **E**, M1 macrophage-related genes expression in *Grn*^−/−^*-*LPS vs. WT-LPS. **F** and **G** represent the *Nlrp3* expression and serum IL-1β in male mice. Data **B** is a Log2-fold change representation of n=3 in each group. Data **C** is presented as mean ± SEM of n=4 mice /group and data **D** includes n=4 for PBS and n=6 for LPS injected mice, respectively, and analyzed using One-way ANOVA with Kruskal-Wallis test. **E** is presented as mean ± SEM of n=3 for each WT and *Grn*^−/−^ female mice. Data **F** (n=3/group) and **G** (n=6/group) are presented as mean ± SEM using One-way ANOVA with Bonferroni’s correction.

Our *in vitro* results suggested that macrophages from *Grn*^−/−^ mice have lower *Nlrp3* expression and possibly reduced NLRP3 inflammasome signaling. To corroborate our *in vitro* findings, we challenged female and male WT and *Grn*^−/−^ mice with LPS (15 mg/kg body wt.) (Fig. 2A). BMMs were prepared and subjected to NanoString-based transcriptome profiling. We observed that female *Grn*^−/−^ BMMs showed differentially expressed genes with more than > 50-fold increase in the expression of *Ccl2* and *Ccl7* (Fig. 2B), which are macrophage chemotactic proteins that also play a role in macrophage polarization. Of interest, *Grn*^−/−^ vs. WT BMM showed reduced expression of *Nlrp3* in response to LPS, which was further validated by qPCR (Fig. 2C). Given that IL-1β production is largely NLRP3 inflammasome dependent, we indeed found that the serum IL-1β was lower in the LPS challanged *Grn*^−/−^ vs.WT counterparts (Fig. 2D). Reportedly, NLRP3 inflammasome signaling is involved in M1-type macrophage polarization. We found that LPS challenged female *Grn*^−/−^ vs. WT BMMs displayed down-regulation of M1-type genes including *iNOS, Tnfa, Fpr2, Cxcl10*, and *Il6* (Fig. 2E). Similarly, a reduced *Nlrp3* expression and less serum IL-1β levels in the *Grn*^−/−^ vs. WT was observed male mice (Fig. 2F and G). Taken together, these results suggest that PGRN expression positively correlates with activation of the LPS-induced NLRP3 inflammasome signaling cascade, which in turn is required for M1-type macrophage polarization.

### Grn ^−/−^ mice show delayed onset of inflammation in response to STA-induced arthritis.

**Figure 3:**
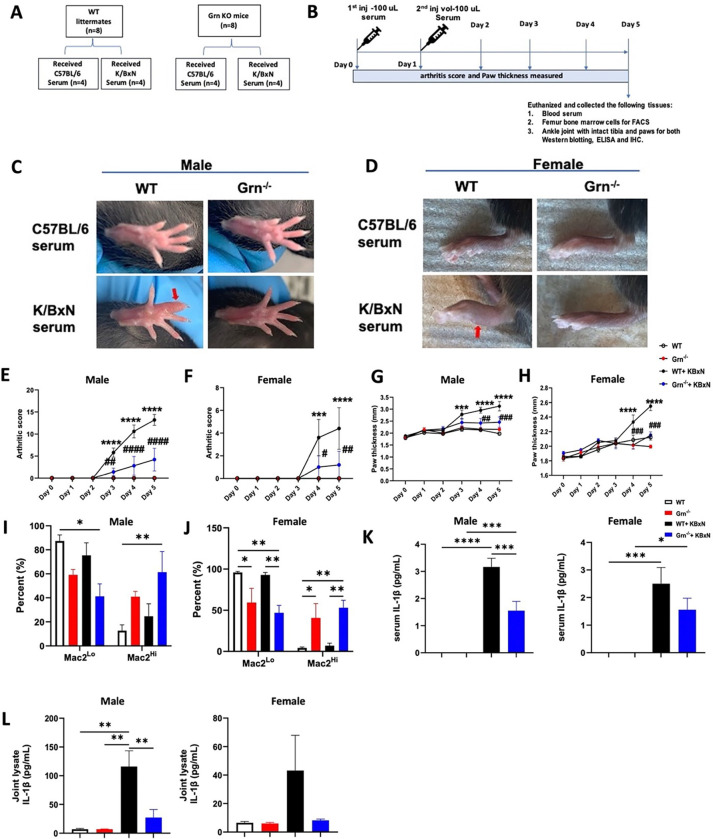
*Grn*^−/−^ mice show decreased acute inflammation induced in the KBxN serum transfer arthritis model. **A**, 2 to 2.5-months-old WT and *Grn*^−/−^ mice were divided in two groups injected with either s C57BL/6 or KBxN serum. **B**, On day 0 and day 1, each mouse was i.p. injected with 100 mL of KBxN or C57BL/6 serum. Paw joint inflammation was assessed in **C,** male and **D**, female mice. Mean arthritis score (MAR) was evaluated in **E**, male and **F**, female mice, and paw thickness (**G, H**) was measured. At the end of the experiment, percentages of Mac2^Lo^ and Mac2^Hi^ BMMs was evaluated in all these experimental groups in both **I**, male and **J**, female mice. IL-1β levels in the **K,** serum and in **L**, paw joint lysate is presented for both male and female mice. Data in **E-J** are presented as mean ± SEM of n= 5 mice/group for males and 4–5 mice/group for females and were analyzed using Two-way ANOVA. Data **K** is presented as mean ± SEM of n=5, 4, 5, 5 (WT, *Grn*^−/−^, WT+KBxN, and *Grn*^−/−^+KBxN, respectively) for males and n=5, 4, 4, 4 for females. Data in **L** is represented as mean ± SEM of n= 5, 4, 5, 5 for males and n= 3/group for female mice. Data in **K** and **L** were analyzed using One-way ANOVA with Bonferroni’s correction.

We investigated the functional relevance of the association of PGRN with NLRP3-inflammasome dependent IL-1β production in the serum-transfer (STA)- induced rheumatoid arthritis (RA) mouse model ^26, 27^. Accordingly, *Grn*^−/−^ and WT mice with KBxN or control serum, and arthritis score and paw thickness was measured from until day 5 (Fig. 3A, B). Both male and female WT mice injected with KBxN serum showed robust inflammation at each time point until day 5 as observed by an increase in mean arthritis score (Fig. 3C-F) and paw thickness (Fig. 3G and 3H). Strikingly, both *Grn*^−/−^ mice showed significantly lower arthritis scores and paw thickness compared to sex-matched WT counterparts (Fig. 3C-H).

To understand the cellular mechanism, freshly isolated bone marrow macrophages were immune-phenotyped on day 5. It was observed that the relative percentages of Mac2^Hi^ and Mac2^Lo^ subsets in both the sexes of WT and *Grn*^−/−^ genotypes remained unchanged, irrespective of whether the mice received control or KBxN serum (Fig. 3I and J). Furthermore, we found that the levels of IL-1β were significantly higher in serum and ankle joint lysates from the WT vs. *Grn*^−/−^ arthritic mice (Fig. 3K and L) and in both sexes. Our data provided important information that in the absence of PGRN both *Nlrp3* gene expression and IL-1β cytokine levels are downregulated consistent with the attenuation of inflammation in STA model of RA in the *Grn*^−/−^ mice. Thus, PGRN likely promotes inflammation via promoting the NLRP3 inflammasome cascade in WT mice subjected to STA induced RA.

### Grn ^−/−^ mice show mitigated inflammation during later stages of STA-induced arthritis and attenuated bone erosion

**Figure 4:**
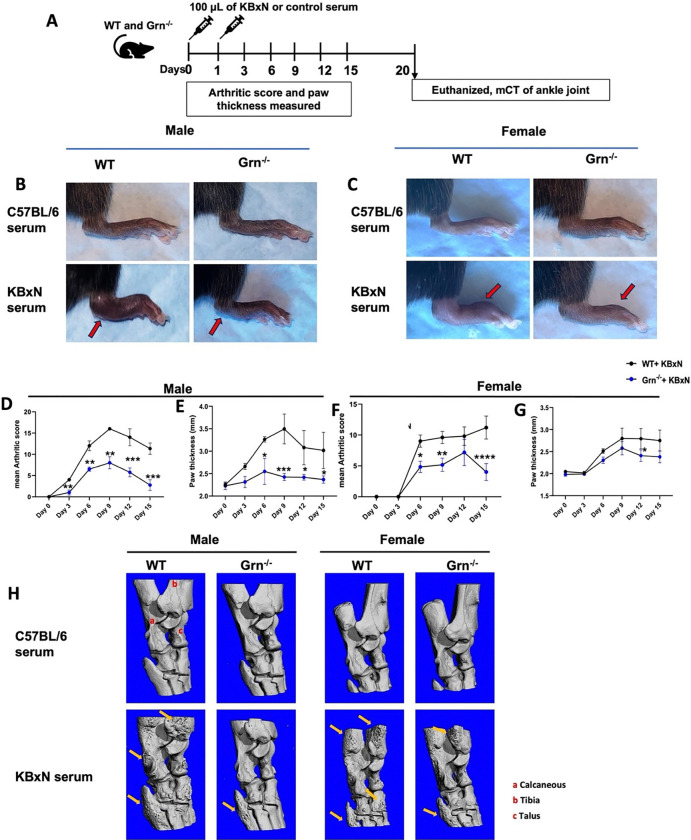
*Grn*^−/−^ mice show mitigated inflammation resulting in reduced severity of bone erosion in response to KBxN serum transfer arthritis model **A**, 2 to 2.5-months-old WT and *Grn*^−/−^ mice were i.p. injected with 100 mL of serum from KBxN or C57BL/6 mice. Paw joint inflammation, as depicted in **B** and **C** for male and female mice respectively was assessed by measuring **D**, mean arthritis score and **E,** paw thickness at the indicated time points in male mice. **F** and **G** show mean arthritis score and paw thickness, respectively, for female mice. On day 20, the ankle joints were subjected to μCT, and **H** shows the representative 3D images of the ankle joints from WT and *Grn*^−/−^ mice from both the sexes treated with C57BL/6 serum or KBxN serum. Data (**D**-**G**) are a representation of mean ± SEM of n= 5 for WT+ KBxN and n=6 for *Grn*^−/−^ + KBxN mice of both the sexes, analyzed using Two-way ANOVA using Bonferroni’s correction.

In STA model of RA, inflammation starts to resolve gradually after day 5–7 and bone erosion is detectable by day 20 ^26, 28^. Therefore, we evaluated the dynamics of inflammation at later stages of RA and bone phenotype as the RA progresses in both the WT and *Grn*^−/−^ mice of both sexes (Fig. 4A). The *Grn*^−/−^ vs. WT mice showed mitigation of inflammation throughout the progression of RA (Fig. 4B and C), evident by a significantly reduction in mean arthritic score and paw thickness (Fig. 4D-G). Furthermore, on day 20, WT arthritic mice showed bone erosion around articular surfaces at the ankle joints, as assessed by μCT (Fig. 4H). Strikingly, arthritic *Grn*^−/−^ mice showed visible protection of erosive bone loss as compared to WT arthritic counterparts (Fig. 4H). In female *Grn*^−/−^ mice, we did see a similar pattern (Fig. 4H). Taken together, these data suggest that PGRN deficiency in mice reduces the severity of erosive bone loss in STA-induced RA.

### Grn ^−/−^ BMMs are highly efferocytic and enhance osteogenic differentiation of bone marrow stromal cells

**Figure 5:**
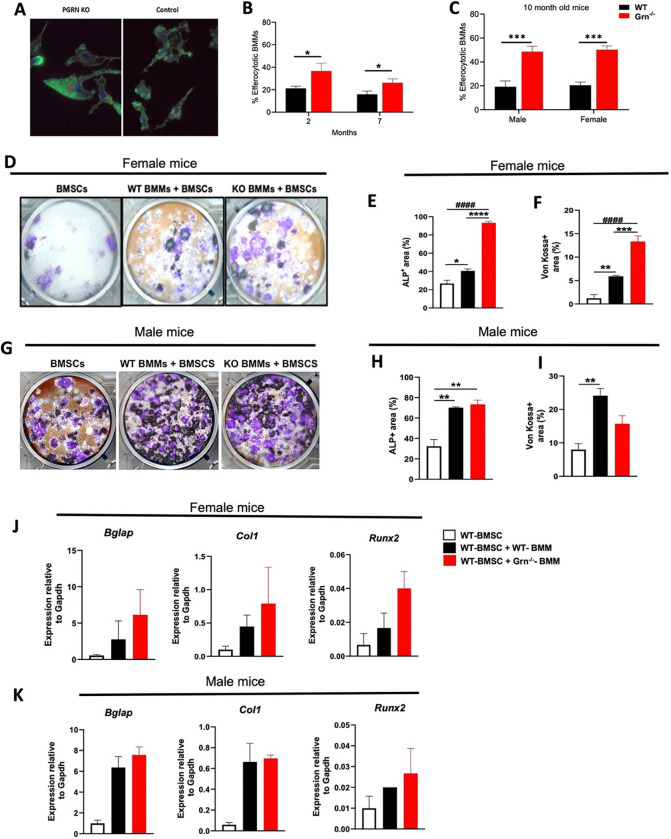
*Grn*^−/−^ macrophages have high efferocytotic potential and display augmented pro-osteogenic function *in vitro* **A**, Representative images of Efferocytic macrophages derived from *Grn*^−/−^ and littermate control (WT) mice (X200). Apoptotic Ocy454 cells were labeled by CellTracker Deep Dye (red). macrophages were labeled in green. **B**, Percentage of efferocytic macrophages derived from both 2- and 7- months old female mice (n= 4 mice/group) were quantified. **C**, the percentage of efferocytic macrophages derived from 10-month-old male (n=4 mice/ group) and female (n=4 mice/ group) mice was quantified. BMMs were isolated from 10-weeks old female WT or *Grn*^−/−^ mice and co-cultured with 6-months-old WT-BMSCs in osteogenic medium. After 21 days, ALP and Von Kossa staining was performed; representative microscopic images are shown in **D** and **G,** quantification is presented in **E** and **F** (female) and **H** and **I** (male) (n=3 mice/group for both the sexes) using Image J software. The expression of osteogenic genes, including *Bglap*, *Col1* and *Runx2* was assessed by qPCR in co-cultures from **J**, female and **K**, male mice (n=3 mice/group). Data in **B** and **C** are presented as mean ± SEM and were analyzed using non-parametric t-test and Two-way ANOVA, respectively. Data in **E**-**K** are presented as mean ± SEM and were analyzed using One-way ANOVA with Bonferroni’s correction.

In the current study, we found that *Grn*^−/−^ BMMs are mostly galectin3^+^ (Mac2^Hi^). Since galectin3 is reported to enhance the efferocytic potential of human monocytes in phagocytosing apoptotic neutrophils ^29^, we assessed the efferocytic potential of PGRN-deficient BMMs. This was achieved by co-culturing labelled apoptotic OCY545 cells with BMMs from either WT and *Grn*^−/−^ mice for 24 hrs and later counting the labelled BMMs (a read out of efferocytosis) using fluorescence microscopy. We found that *Grn*^−/−^ BMMs showed a higher percentage of labeled cells (red) due to increased efferocytosis of apoptotic OCY545 as compared to WT BMMs at 2 months and 7 months of age, and in both the sexes (Fig. 5A-C). These results suggest that PGRN limits macrophage efferocytic potential, possibly via downregulation of Mac2 expression.

It is well established that osteal macrophages are critical to bone formation via different mechanisms, including depletion of apoptotic osteoblasts and by the recruitment of osteoblast precursors ^30^. Also, the osteogenic differentiation of bone marrow stem cells is influenced by osteal macrophages and further influenced by macrophage phenotype, with M1 being inhibitory and M2 with stimulatory actions ^31^. We assessed the potential of PGRN deficient BMMs to regulate bone marrow stromal cell osteogenic differentiation. Female WT BMSC co-cultured with WT BMMs displayed enhanced ALP^+^ area and Von Kossa^+^ area as compared to the control WT BMSC alone cultures (Fig. 5D). However, intriguingly, female *Grn*^−/−^ BMMs co-cultured with WT-BMMs exhibited an increased ALP^+^ and Von Kossa^+^ areas compared with either the WT BMM + WT BMSC co-culture or WT BMSC alone cultures (Fig. 5D, E and F). This suggests that BMMs from female *Grn*^−/−^ mice have greater pro-osteogenic activity than BMMs from female WT mice. In the co-culture experiments using male mice, WT BMMs did enhance the osteogenic differentiation of BMSCs as compared to BMSC cultures with no BMMs added to them, as assessed by the increase in the percent ALP^+^ and Von Kossa^+^ areas (Fig. 5D, H and I). However, BMMs from male *Grn*^−/−^ mice did not display enhanced pro-osteogenic activity compared to BMMs from male WT mice. We further confirmed these observations using qPCR-based relative gene expression of osteogenic marker genes, including *Alp, Runx2, Col1, and Ocn.* Gene expression studies of the co-cultures clearly showed that female *Grn*^−/−^ BMM have higher pro-osteogenic activity than the WT-BMMs (Fig. 5J), while male *Grn*^−/−^ BMMs did not further enhance the expression of osteogenic genes as compared to BMM from WT male counterparts (Fig. 5K). Taken together, these results suggest that the PGRN deficient BMMs derived from female *Grn*^−/−^ mice have greater pro-osteogenic supporting potential than WT BMMs or male *Grn*^−/−^ BMMs.

### Female Grn^−/−^ mice display greater bone anabolic action of iPTH in vivo

**Figure 6:**
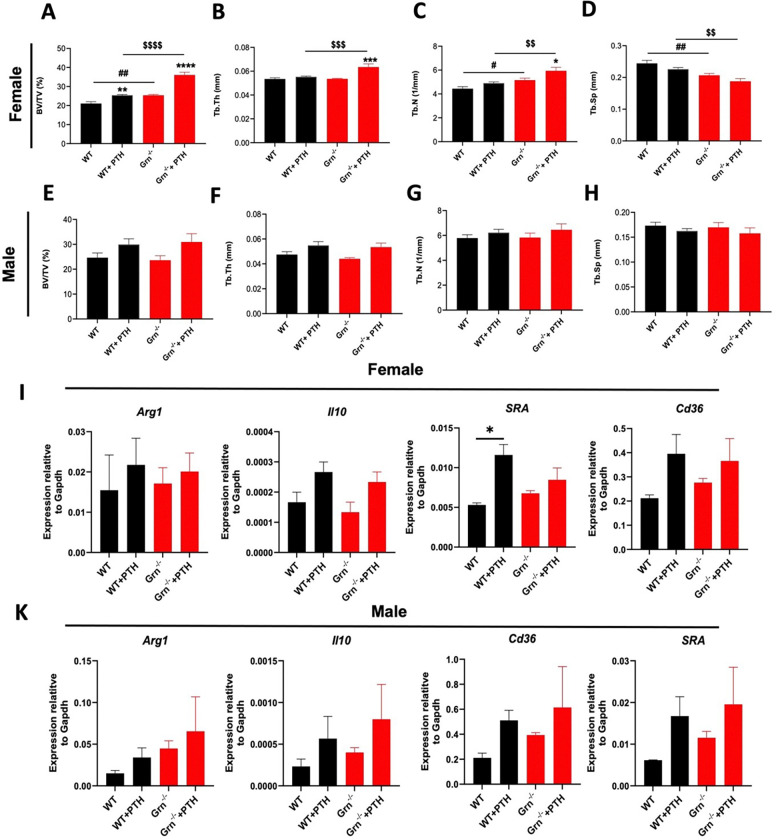
*Grn*^−/−^ female mice display greater anabolic effects of iPTH on vertebral trabecular bone 4-months old male and female *Grn*^−/−^ and WT control littermates were s.c. injected with rh PTH or solvent vehicle for five consecutive days per week, for 4 weeks. *In vivo* mCT assessment of cancellous bone at L5 vertebral bone in (**A-D**) female (n=7 WT, n= 8 *Grn*^−/−^, n=6 WT+ PTH, and n= 5 *Grn*^−/−^+ PTH) and (**E-H**) male mice (n=6 WT, n= 7 *Grn*^−/−^, n=5 WT+ PTH, and n= 6 *Grn*^−/−^+ PTH) were performed. Bone fractional volume, BV/TV; Tb.N, trabecular number; Tb.Th, Trabecular thickness; Tb.Sp, trabecular separation. BMMs were harvested from these mice and the expression of efferocytic macrophage marker genes, including *Arg1, Il10, Cd36,* and *SRA* were assessed by qPCR (n=3 mice/group for both the sexes). Data are presented as Mean ± SEM analyzed using One-way ANOVA post-hoc Tukey’s correction for **A**-**H** and Two-way ANOVA post-hoc Boneferroni’s correction for **I** and **K**. * p < 0.0332, ** p < 0.0021, *** p < 0.0002, **** p < 0.0001 vs. vehicle-treated WT or *Grn*^−/−^.

In our current study, we observed that BMMs derived from female *Grn*^−/−^ mice showed greater pro-osteogenic function in driving the osteogenic differentiation of BMSCs. Also, it has been reported that macrophages are essential for bone anabolic actions of iPTH therapy in mice ^32^. Thus, we evaluated whether PGRN-deficient macrophages are different in responses to iPTH therapy-induced bone anabolic actions *in vivo* and if sex-differences are observed. well. To achieve this, we s.c. injected both WT and *Grn*^−/−^ mice with PTH (80 μg/kg/day) for 5 days/ week for 4 weeks. The L_5_ vertebrae were subjected to bone phenotyping using mCT after 4 weeks of PTH treatment. We observed that administration of PTH induced bone formation in both male and female WT mice and *Grn*^−/−^ mice as shown by a significant increase in BV/TV, Tb.Th., and Tb.N. and a decrease in Tb.Sp. (Fig. 6A-H). Notably, PTH-injected *Grn*^−/−^ female mice (*Grn*^−/−^+PTH) had an increased anabolic response to iPTH compared to their female WT counterparts injected with PTH (WT + PTH), as shown by a higher BV/TV, Tb.Th., and Tb.N. with a significant decrease in Tb.Sp. (Fig. 6A-D). However, PTH-injected *Grn*^−/−^ male mice (*Grn*^−/−^+PTH) showed similar parameters of trabecular bone to male WT counterparts injected with PTH (WT + PTH). These data are consistent with our *in vitro* findings of the pro-osteogenic function of PGRN-deficient macrophages. Furthermore, immunostaining of F4/80^+^ osteal macrophages on the femur bone surface of the WT and *Grn*^−/−^ mice did not show any difference in osteal macrophage number in any of the groups or between the sexes (Suppl. Figure 1). Moreover, BMMs derived PTH injected mice, both female and male exhibited a pattern of increased expression of efferocytic macrophage marker genes, including *Arg1*, *Il10*, *Cd36* and *SRA*, irrespective of the sex and genotype.

### Transcriptomic profiling of female Grn^−/−^ bone marrow stromal cells (BMSCs) reveal downregulation of inhibitory Gi signaling pathway

**Figure 7:**
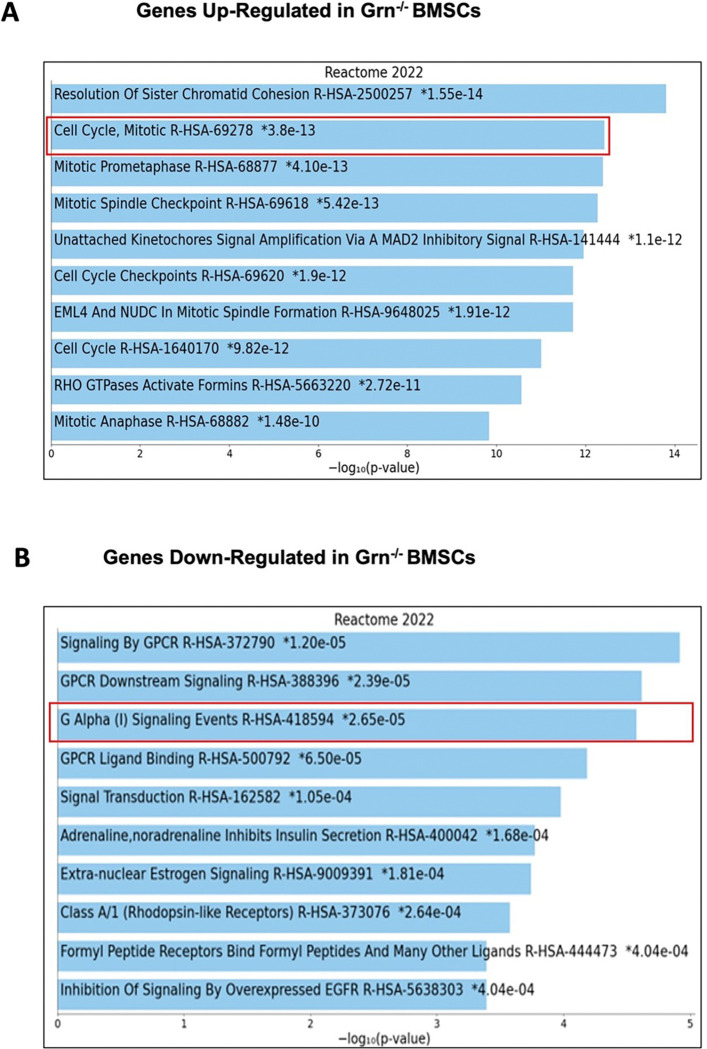
Transcriptomic profiling of *Grn*^−/−^ bone marrow stromal cells (BMSCs) reveal upregulation of cell cycle pathway and downregulation of inhibitory Gi signaling pathway *Grn*^−/−^ and WT -BMSCs (n=3 mice/ group) were allowed to undergo osteogenic differentiation for 21 days and total RNA was subjected to bulk RNAseq analysis followed by REACTOME analysis shows **A**, up-regulated and **B**, down-regulated pathways in *Grn*^−/−^-BMSCs as compared to WT-BMSCs.

In the present study, we found that female *Grn*^−/−^ BMMs have greater potential of promoting osteogenic differentiation of BMSCs *in vitro*, and *in vivo* PTH stimulated higher bone anabolic effects in female *Grn*^−/−^ mice. Thus, we performed RNAseq on the BMSCs after 21 days of osteogenic differentiation from both WT and *Grn*^−/−^ to understand any intrinsic differences. We found that *Grn*^−/−^ BMSCs showed > 5-fold increase in expression of genes of cell cycle progression, and > 3-fold decrease in the expression of genes that belong to inhibitory Gi signaling. Further Reactome analysis indeed showed upregulation of cell cycle genes and down-regulation of genes involved in Gi signaling in the *Grn*^−/−^ BMSCs vs. WT-BMSCs (Fig. 7A, B). We know from our previous work that inhibition of Gi signaling with pertussis toxin, using genetic mouse models, enhances bone formation in aging females, and accelerates bone anabolic action of PTH only in female mice ^33, 34^. The resulting active Gsα signaling pathway, which is a known PTH target, mediates the bone formation function of osteoblasts.

## DISCUSSION

Available reports indicate PGRN has dual action, acting as a pro- or anti-inflammatory molecule depending on the cellular context ^35^. Full-length PGRN can be digested by proteases, both intracellularly or extracellularly, into different granulins including, G, F, B, A, C, D, and E, which are known to play proinflammatory roles ^12^. Reportedly, PGRN is anti-inflammatory in mouse models of osteoarthritis (OA) and collagen-induced arthritis (CIA) and is attributed to its potential to block the TNF-ɑ pathways by blocking its receptors (TNFRs) ^14, 15^. Similarly, Attstrin, PGRN analog, is anti-inflammatory ^8^. In addition to its anti-inflammatory role, PGRN is essential for efficient osteoclast differentiation as the *Grn*^−/−^ OC precursors are resistant to RANKL- induced osteoclast formation *in vitro* ^34^. Additionally, PGRN protects from the inhibitory action of TNF-α on osteoblasts differentiation ^22^. Previously, we reported that female *Grn*^−/−^ are resistant to aging-associated bone loss. This is partly attributed to higher bone formation rate and reduced osteoclastic bone resorption. However, male *Grn*^−/−^ mice do not exhibit such an aging phenotype and continue to lose bone despite reduced osteoclastic bone resorption. However, the mechanism(s) underlying the female-sex specific aging-associated bone protective role of PGRN is not clear. The female-specific bone protective role of PGRN was also seen in *Cx3Cr1*^*Cre*^; *Grn*^*f/f*^ mice, suggesting that the negative role of PGRN on bone mass results from production of the protein by macrophage lineage cells. Thus, in the present study we investigated the role of PGRN in mediating the effects of macrophages on bone homeostasis.

Firstly, to understand the role of PGRN in macrophage biology, we characterized freshly isolated bone marrow macrophages ^25^. We found that *Grn*^−/−^ mice have a higher percentage of Mac2^Hi^ macrophages in both male and female mice, suggesting that PGRN may have a role in the regulation of Mac2 expression. Mac2, also known as galectin3, is upregulated in microglia of patients with haploinsufficiency due to loss-of-function mutation in *Grn* genes. Galectin3 has been reported to be essential for human macrophage invasion and for suppressing pro-inflammatory cytokine production ^36^. Interestingly, our transcriptomics data indeed revealed that Mac2^Hi^ macrophages express low levels of inflammation-related genes, and notably, we found that *Nlrp3* was downregulated in these PGRN-deficient macrophages. As NLRP3 inflammasome signaling is involved in the maturation and secretion of IL-1β, we found that the serum IL-1β levels and *Nlrp3* expression in the M-CSF dependent BMMs were lower in the *Grn*^−/−^ mice as compared to the WT in response to LPS challenge, thus suggesting that PGRN expression is positively correlated with NLRP3 inflammasome signaling pathway gene expression.

To understand the disease relevance of these findings, we employed a serum transfer-induced rheumatoid arthritis (RA) model. This model of RA is principally dependent on IL-1β-mediated inflammation and bone loss. The results clearly indicated that PGRN presence is required for effective NLRP3 mediated-IL-1β-induced inflammation as the *Grn*^−/−^ showed signs of alleviated inflammation in the paw in both the sexes and that the mice showed reduced severity of bone erosion. The reduced serum and paw joints levels of IL-1β in *Grn*^−/−^ further suggests that PGRN regulates NLRP3 inflammasome signaling leading to IL-1β-mediated inflammation, arthritis and the subsequent bone erosion in WT mice.

In the present study, M-CSF dependent BMMs from both male and female *Grn*^−/−^ mice demonstrated enhanced efferocytotic activity *in vitro*. Increased efferocytotic activity of macrophages has been reported to be associated with increased bone formation *in vivo* ^30^, which is due to clearance of apoptotic mature osteoblasts and recruiting new osteoblast precursors at the site of bone formation. These efferocytic macrophages are reported to be F4/80^+^ osteomacs present on the bone surface. Further, Cho et al. in 2014 reported that these F4/80^+^ osteomacs are critical for the anabolic action of intermittent parathyroid hormone therapy (iPTH) ^32^. Interestingly, we found that BMMs from female *Grn*^−/−^ co-cultured with bone marrow stromal cells (BMSCs) from WT mice showed greater osteogenic differentiation potential than the WT BMMs or BMMs alone However, BMMs from male *Grn*^−/−^ co-cultured with bone marrow stromal cells (BMSCs) did not show enhanced osteogenic differentiation from WT counterparts. Further, *in vivo*, we found that the female *Grn*^−/−^ mice showed enhanced anabolic action of iPTH in L5 vertebrae as compared to their WT counterparts. These results further highlight the importance of macrophage efferocytotic function as an essential process of iPTH therapy. However, for reasons unknown, in our study the enhanced anabolic effect of iPTH was limited only to female *Grn*^−/−^ despite increased efferocytic function in both the sexes *in vitro*. Further, the transcriptomics and reactome analysis of BMSCs from *Grn*^−/−^ revealed downregulation of genes involved in the Gi signaling pathway. Intriguingly, Gi signaling has been well documented to hamper bone formation and to limit the anabolic action of iPTH therapy in female mice ^33,34^. One such Gi-GPCR is *Htr1b* which was down-regulated in the female *Grn*^−/−^ BMSCs. It is well-documented that gut-derived serotonin binds to *Htr1b* on osteoblasts and inhibits bone formation via decreasing cAMP response element-binding protein (CREB) function, a key transcription factor that promotes osteoblast proliferation and differentiation ^37^. Thus, an increased pro-osteogenic and efferocytic functions of macrophages together with inhibition of Gi signaling in *Grn*^−/−^ females can partly explain the enhanced bone anabolic action of iPTH therapy. This could possibly explain our previous finding that only female *Grn*^−/−^ mice are resistant to aging-associated bone loss ^34^. However, further mechanistic studies are required to understand the sexual dimorphic actions of PGRN on aging-induced bone loss.

In summary, deficiency of PGRN expression in mouse macrophages downregulates *Nlrp3* and *Il1β* expression to alter NLRP3 signaling cascade which confers macrophage with less inflammatory phenotype. Consequently, the severity of bone erosion is reduced in *Grn*^−/−^ serum transfer-induced RA model in mice. Furthermore, these PGRN-deficient macrophages exhibit enhanced osteogenic potential that can contribute to greater anabolic action of PTH in *Grn*^−/−^ female mice and protect these mice from aging-associated bone loss. However, molecular mechanisms governing progranulin-mediated activation of the NLRP3 inflammasome and its bone anti-anabolic effects are unclear and will be of great interest for further studies.

## MATERIALS AND METHODS

### Animals

All animal studies were approved by and performed in accordance with the Institutional Animal Care and Use Committees at the San Francisco VA Medical Center and the University of California, San Francisco (UCSF). We bred heterozygous *Grn*^*−/+*^ mice in C57BL/6 background, generously provided by Dr. Robert V. Farese at UCSF, to generate *Grn*^−/−^ and WT littermate (used as controls). KRN mice, provided by Dr. Clifford Lowell at UCSF, were bred with NOD mice (The Jackson Laboratory, Bar Harbor, ME) to generate K/BxN mice. *Grn*^−/−^ and WT mice of both sexes and various ages were used in different experiments.

### Isolation of mouse bone marrow-derived macrophage and culture of M-CSF dependent macrophages (BMMs)

Freshly isolated bone marrow macrophages were immune-phenotyped (flow cytometry section) or cultured with M-CSF to generate M-CSF-dependent macrophages (BMMs) ^25^. Briefly, mice were euthanized and hindlimb bones were excised, demuscled, and the epiphyseal ends were cut open, and the marrow was flushed with RPMI-1640 (Gibco) using a syringe with a needle size of 26^1/2^ gauge. Upon RBCs lysis using Lysis Buffer (Cat#00-4333-57, eBioscience), cells were washed and resuspended in RPMI-1640 growth medium containing 10% FBS, 1% penicillin-Streptomycin, and 0.1% Fungizone, and 20 ng/mL macrophage-colony stimulating factor (M-CSF; Cat# 416-ML-050/CF, R&D Systems) for 24 hours. Next day, the non-adherent fraction is collected and cultured in RPMI-1640 growth medium with M-CSF (20 ng/mL) for 6 days to generate macrophages (BMMs).

To understand the effect of PGRN deficiency or action of exogenous PGRN on the expression of the proinflammatory genes, BMMs were prepared from 12-weeks-old *Grn*^−/−^ and their littermate WT. The BMMs were treated for 24 hrs with 500 ng/mL of recombinant mouse PGRN (Cat# AG-40A-0189Y-C010, AdipoGene Life Sciences).

### Bone marrow stromal cells (BMSCs) isolation and co-culture with BMMs

Bone marrow macrophages (BMMs) were prepared from 10-month-old *Grn*^−/−^ or WT mice as described above. On the day before the co-culture assay, male and female C57BL/6 mice of 10 months age were used for isolating bone marrow stromal cells (BMSCs). In brief, total bone marrow was flushed out and BMSCs were enriched with MACS technology by depleting the mature hematopoietic lineage cells with CD11b^+^ MACS microbeads (Cat#130-126-725, Miltenyi Biotec). The enriched BMSCs were plated in a 6-well plate at a density of 3 × 10^6^ cells/well for female cell BMSCs and 2.6 × 10^6^ cells /well for male BMSCs (day 0). On day 1, bone marrow macrophages (BMMs) were enzymatically freed and then seeded into the culture wells containing BMSCs in a ratio of 1:7 (BMMs/BMSCs). The cultures were maintained undisturbed for 5 days in a 5% CO_2_ maintained at 37°C. culture medium was removed along with all non-adherent cells and replaced with fresh alpha MEM with 50 μg/ml ascorbic acid and 3 mM β-glycerophosphate to initiate osteogenic differentiation. The culture medium was replaced every three days. At day 21, alkaline phosphatase and Von Kossa stainings were performed using ALP staining kit (Cat# ab284936, Abcam) and Silver Nitrate staining methods, respectively. Stained areas were quantified using NIH Image J software.

### RNA isolation, cDNA preparation and qPCR

Total RNA was isolated from the cells using TRI reagent-based phenol-chloroform isolation followed by purification with RNeasy mini kit (Cat# 74104, Qiagen), according to the manufacturer’s instructions. The isolated Total RNA was used to prepare cDNA using TaqMan^™^ Reverse Transcription Reagents (Cat# N8080234, Thermo-Fisher) according to the manufacturer’s instructions. qPCR was performed using 10 ng of cDNA using SYBR^™^ Green Universal Master Mix and 100 nM of forward and reverse primer pairs for each gene, designed using Primer Bank. *Gapdh* was used as an endogenous control gene, and the relative expression of the gene was calculated using 2^− dCT^. Primers used for all genes are listed in Table 1.

### Flow cytometry

Freshly isolated adult mouse bone marrow macrophages were characterized as described previously ^25^. In brief, freshly isolated bone marrow cells after RBS lysis were incubated with TruStain FcX to block the Fc receptors and cells were stained with fluorescence tagged antibodies as listed in Table 2. BD compensation beads were used for setting up compensation. Samples were acquired using BD FACSAria^™^ FUSION and populations were sequentially gated via FMOs.

### NanoString nCounter

7-months-old male and female C57BL/6 mice were used to evaluate the differential transcriptome of Mac2^Lo^ vs Mac2^Hi^ in freshly isolated macrophages population. In brief, freshly isolated mouse macrophages were enriched with CD11b^+^ MACS microbeads, incubated with Fc block, and then stained for Mac2 antibody (Table 2) to sort Mac2^+^ cells with high (Mac2^Hi^) and low Mac2 (Mac2^Lo^) with a BD FACSAria^™^ FUSION at the SF VAMC FACS core. RNA was extracted from the sorted Mac2^Lo^ and Mac2^Hi^ cells with an Invitrogen PureLink RNA Micro Scale kit (Waltham, MA). Similarly, RNA from M-CSF dependent BMMs harvested from LPS-challenged WT and *Grn*^−/−^ were subjected to NanoString nCounter system (Seattle, WA) using a nCounter^®^ Myeloid Innate Immunity Panel.

### LPS challenge experiment

Male and female *Grn*^−/−^ and WT littermates of 6–9-months-age were i.p. injected with 15 mg/kg Lipopolysaccharide (LPS) (Cat# L4130, Escherichia coli O111:B4, Sigma, MO). After 6 hrs, the mice are euthanized, bone marrow was collected and M-CSF dependent BMMs were prepared. Subsequently, BMMs were subjected to NanoString-based gene expression analysis. Blood serum was also collected for cytokine level measurement.

### Induction of serum-transfer-induced (STA) rheumatoid arthritis

We bred the KRN mice with NOD mice to obtain the K/BxN mice. The blood is collected from K/BxN mice at the age of 8–10 weeks old and allowed to clot at RT for 15 min and then centrifuged at 2000 × g for 10 min at 4°C. The KBxN serum is then stored in −80 until use. We used the serum from C57BL/6 mice of the same age as control serum.

The experiment involved injection of 100 μL of K/BxN serum or control serum in 2- months-old WT and *Grn*^−/−^ male and female mice. Two injections were done one on day 0 and on day 1 i.p. As a measure of inflammation, arthritis score and paw thickness were measured on each day until day 5 post first injection for the inflammatory phase study. For the effector phase study, the inflammation was monitored every three days until day 15 ^26, 28^. After euthanizing the mice, serum was collected, one ankle joint from each mouse was snap frozen for ELISA. The femurs were used to characterize the BMMs using flow cytometry.

### Measurement of arthritis score and paw thickness

To measure inflammation in the RA mice, we used the arthritis scoring method as previously described ^26, 28^. For each of the four limbs (maximum four points per limb, up to a combined total of 16), score points (1–4) according to the presence of the feature with the greatest point value.1 point if there is only redness of the bottom of the footpad; 2 points if there is visible thickening of the paw, 3 points if the swelling of the ankle is sufficient to make the ankle equal to or greater in width than the mid footpad, 4 points if there is swelling of at least one digit. The paw thickness was measured using digital calipers at the ankle joint (malleoli) and is presented in millimeters (mm).

### Intermittent Parathyroid hormone (iPTH) administration

4-months-old WT and *Grn*^−/−^ mice of both the sexes were injected s.c. with recombinant human PTH 1–34 (Bachem Inc., CA) (dissolved in 10 mM acetic acid in PBS with 2% heat inactivated C57BL/6 serum) at a dose of 80 μg/kg body weight for five consecutive days per week, for 4 weeks. Control animals were treated with the same volume of vehicle – 10% acetic acid PBS in PBS containing 2% heat-inactivated C57BL/6 serum. At the end of the experiment, mice were euthanized and L5 vertebrae and hind limbs were fixed in 4% PFA and then subjected to micro-CT (μCT) analysis for bone phenotyping.

### Micro-computed Tomography

Hind limb paws with intact ankle joint from the RA mice and L5 vertebra from PTH-treated mice were fixed in 4% PFA for 48 hours at 4°C and then stored in 70% ethanol before being assessed using μCT scan and histomorphometry. Ankle joints were scanned using a Scanco VivaCT-50 μCT system (Scanco Medical, Brüttisellen, Switzerland) with an X-ray energy of 55 kV, a voxel size of 10.0 μm, and an integration time of 500 ms. We analyzed the 3D images of the whole ankle joint and visually assessed the bone erosion. For the PTH study, the trabecular region of interest (ROI) within L_5_ was assessed. The ROI was defined as a cylindrical volume of 0.5 mm^2^ cross-sectional area and trabecular parameters were evaluated.

### Serum and paw joint IL-1β measurement

To assess the levels of cytokines in the serum in LPS-injected animals, we used a flow cytometry-bead based immunoassay using LegendPlex^™^ Mouse M1 Macrophage Panel (8-plex) (Cat#740848, Biolegend), according to the kit instructions. The concentration of each cytokine is subsequently determined by referencing a standard curve generated concurrently within the same assay using online LEGENDplex^™^ Data Analysis Software by applying a 5-parameter curve fitting algorithm. For serum-transfer induced arthritis studies, serum and paw-joint lysate IL-1β was measured using Mouse IL-1 beta/IL-1F2 DuoSet ELISA kit (Cat# DY401-05) following manufacturer’s instructions.

### Immunofluorescence

Tibiae were collected from PTH or vehicle treated WT and *Grn*^−/−^ mice and fixed in 4% PFA followed by demineralization. 5 μm thick sections were made using cryotome and subjected to IHC. Briefly, the sections were blocked with 5% donkey serum in PBS and then incubated with primary antibody against F4/80 (Cat# MF48000) overnight at 4^ο^C in a humidified chamber. Next day, the sections are washed and incubated with Goat anti-rat Alex Flour^™^ 555 (Cat# A-21434). The slides were analyzed at 20X magnifications using the microscope BZ-X800 (Keyence).

### RNAseq

BMSCs from WT and *Grn*^−/−^ female mice (n = 3/genyotype) underwent osteogenic differentiation as described before. At day 21, RNA was isolated and RNAseq analysis was performed by Novogene using NovaSeq PE150 platform. Pathway analysis was carried out using the Reactome database and differentially regulated genes were assessed using threshold is normally set as: p adj < 0.05.

### Statistical analysis

Statistical analysis was carried out using Graph pad Prism 10 software (version 10.6.1). For comparison of two groups, we used an unpaired student’s t-test. For experiments with more than 2 groups, we used one-way ANOVA with post-hoc Bonferroni’s correction and for more than 2 parameters, we used Two-way ANOVA. Each graph presented is a mean ± SEM.

## Supplementary Material

Supplementary Files

This is a list of supplementary files associated with this preprint. Click to download.

- SupplementaryFig.docx

## Figures and Tables

**Table 1:** List of all qPCR primers

Primer	Forward Primer (5’–3’)	Reverse Primer (5’–3’)
*Nlrp3*	AGA AGA GAC CAC GGC AGA AG	CCT TGG ACC AGG TTC AGT GT
*Il1β*	GTG CAA GTG TCT GAA GCA GC	CAA AGG TTT GGA AGC AGC CC
*Tnfα*	GCC TCC CTC TCA TCA GTT CTA	GGC AGC CTT GTC CCT TG
*Il6*	ATC CAG TTG CCT TCT TGG GAC TGA	TAA GCC TCC GAC TTG TGA AGT GGT
*iNOS*	GAG ACA GGG AAG TCT GAA GCA C	CCA GCA GTA GTT GCT CCT CTT C
*Fpr2*	GAG CCT GGC TAG GAA GGT G	TGC TGA AAC CAA TAA GGA ACC TG
*Cxcl10*	CCA AGT GCT GCC GTC ATT TTC	GGC TCG CAG GGA TGA TTT CAA
*Bglap*	CTG ACC TCA CAG ATG CCA AG	GTA GCG CCG GAG TCT GTT C
*Col1*	GCG AAG GCA ACA GTC GCT	CTT GGT GGT TTT GTA TTC GAT GAC
*Runx2*	CGA GAC CAA CCG AGT CAT TT	ACG CCA TAG TCC CTC CTT TT
*Arg-1*	TGT CCC TAA TGA CAG CTC CTT	GCA TCC ACC CAA ATG ACA CAT
*Il10*	CTG GAC AAC ATA CTG CTA ACC G	GGG CAT CAC TTC TAC CAG GTA A
*Cd36*	ATT AAT GGC ACA GAC GCA GC	GCA TTG GCT GGA AGA ACA AA
*SRA*	GTC GGG ATC TCC TGG ACC TA	ATC CCA GCG ATC ATC ACA GA
*Gapdh*	TGC ACC ACC AAC TGC TTA G	GGA TGC AGG GAT GAT GTT C

**Table 2:** List of antibodies used for flow cytometry

Antibody	Catalog	Company
TruStain FcX (Clone 93)	101320	Biolegend
Pacific Blue anti-mouse CD45 (clone 30-F11)	103126	Biolegend
Allophycocyanin (APC) F4/80 (clone BM8)	123116	Biolegend
APC-Cy7 rat anti-CD11b (clone M1/70	557657	Biolegend
PE/Dazzle 594 Ly-6G (clone1A8)	127648	Biolegend
Spark UV^™^ 387 anti-mouse Ly-6C Antibody (clone HK1.4)	128059	Biolegend
Alexa Fluor 488 Mac2 (clone M3/38)	125410	Biolegend

## Data Availability

All datasets generated during and/or analyzed during the current study are presented in the article. Any additional raw files of transcriptomics are available from the corresponding author on reasonable request.
